# Long‐Term Use of Benzodiazepines and Related Drugs in Persons With Major Depressive Disorder

**DOI:** 10.1002/brb3.71085

**Published:** 2025-11-17

**Authors:** Heli Laitinen, Miia Tiihonen, Antti Tanskanen, Jari Tiihonen, Johannes Lieslehto, Marjaana Koponen, Heidi Taipale

**Affiliations:** ^1^ School of Pharmacy University of Eastern Finland Kuopio Finland; ^2^ Department of Forensic Psychiatry University of Eastern Finland, Niuvanniemi Hospital Kuopio Finland; ^3^ Department of Clinical Neuroscience Karolinska Institutet Stockholm Sweden; ^4^ Center for Psychiatry Research Karolinska Institutet, Stockholm City Council Stockholm Sweden; ^5^ Institute for Molecular Medicine Finland University of Helsinki Helsinki Finland

**Keywords:** benzodiazepines, depression, insomnia, pharmacoepidemiology

## Abstract

**Introduction:**

Benzodiazepines and related drugs (BZDRs) may be prescribed to treat anxiety and insomnia in persons with depression. Although BZDRs are recommended for short‐term use only, their use may be prolonged. Despite research on the use of BZDRs in depression, to our knowledge, long‐term use and its predictors have not been previously studied. The aim of this study was to estimate the incidence of long‐term BZDR use in persons with depression and to identify sociodemographic and clinical factors associated with prolonged use.

**Methods:**

Data were extracted from Finnish nationwide registers. The study focused on persons with depression aged 16–65 years who initiated BZDR use between July 1, 2015 and June 30, 2018. Persons with a previous diagnosis of bipolar disorder, schizophrenia‐spectrum disorder, or dementia, or those who did not have at least 180 days of follow‐up, were excluded from the study. Sociodemographic and clinical factors associated with long‐term (≥180 days) versus shorter use were compared with logistic regression. The final study sample included 11,303 BZDR initiators.

**Results:**

A total of 849 (7.5%, CI 95% 7.0–8.0) BZDR initiators became long‐term users. The mean age of long‐term users was 39.8 years (SD 13.3), and more than half of them were females (58.9%). Factors associated with long‐term BZDR use included age over 45 years (45–54: aOR 1.56, 95% CI 1.22–1.99, ≥55: 1.72, 1.32–2.25 compared to ≤24 years), male gender (1.49, 1.28–1.73), substance use disorder (excl. alcohol) (1.79, 1.27–2.29), use of opioid (1.75, 1.38–2.21) or quetiapine (1.65, 1.18–2.27), ≥3 antidepressants used in the previous year (1.63, 1.22–2.19), socioeconomic status other than employed, the use of antidepressants with hypnotic effects (1.25, 1.06–1.47) and anxiety disorder (1.16, 1.00–1.35).

**Conclusions:**

This Finnish population‐based cohort study identified sociodemographic and clinical factors that should be considered in the initiation and monitoring of BZDR treatment among persons with depression.

## Graphical abstract

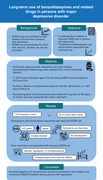

## 1 Introduction

Depression is a common mental disorder and it is estimated that around 5% of adults worldwide suffer from depression (World Health Organization 2023). In the Finnish population, annual prevalence in adults is approximately 5%–7%, with slightly lower estimates reported in people aged over 65 years (Finnish Medical Society Duodecim and Finnish Psychiatric Society 2025). The prevalence of depression is about 1.5–2 times higher in females than in males.

Depression and insomnia have been identified as having a two‐way association and this relationship has been studied in different ways in the general population. Insomnia is one of the known risk factors for depression (Ohayon 2002, Baglioni et al. 2011, Li et al. 2016, Fang et al. 2019, Morin et al. 2024). Around 25% of people who experience insomnia have also reported depression (Oh et al. 2019). On the other hand, many people with depression experience insomnia (Sunderajan et al. 2010, O'Brien et al. 2011, Finnish Medical Society Duodecim and Finnish Psychiatric Society 2025). Insomnia symptoms have been found to be present in about 80% of people with depression (Ohayon 2002). Non‐pharmacological psychosocial treatments, such as Cognitive Behavioral Therapy for insomnia (CBT‐i) are considered the first‐line treatment for insomnia (Finnish Medical Society Duodecim and Finnish Sleep Research Society 2023, Morin et al. 2024). However, pharmacological options, such as antidepressants with hypnotic effects, melatonin, or short‐term use of benzodiazepines or related drugs (BZDR), are often used for treating insomnia. BZDRs for short‐term use may be prescribed to treat insomnia in persons suffering from depression (Finnish Medical Society Duodecim and Finnish Sleep Research Society 2023, Riemann et al. 2023, Morin et al. 2024).

Meta‐analyses show that BZDRs have a positive effect on insomnia when they are used for up to four weeks (Riemann et al. 2023), as they reduce sleep onset latency and increase total sleep time (Finnish Medical Society Duodecim and Finnish Sleep Research Society 2023). BZDRs have a wide range of adverse effects, including the development of tolerance and dependence, daytime drowsiness, nocturnal confusion, risk of falls and motor vehicle accidents, negative effects on cognitive function such as memory decline, as well as rebound insomnia following discontinuation of the drug (Capiau et al. 2022, Riemann et al. 2023, Morin et al. 2024). The use of BZDRs may become prolonged, although they are only recommended for short‐term use. A previously published nationwide Finnish study reported that in 2014, about 9.3% of the Finnish adult population used BZDRs (*n* = 408,527) and 3.6% of the total adult population were long‐term users (*n* = 159,239) (Kurko et al. 2018).

The prevalence of BZDRs use has been studied previously in persons with depression (Nygren et al. 2024). However, to our knowledge, previous studies have not assessed patterns of long‐term use or the predictors of long‐term use. Long‐term use of BZDRs has been studied previously in the general population and, for example, among older people, but not specifically in people with depression (Kurko et al. 2015, Kurko et al. 2018, Taipale et al. 2020, Rosenqvist et al. 2024). The aim of this study was to assess the incidence of long‐term BZDR use in persons with depression and the sociodemographic and clinical factors associated with long‐term use.

## 2 Materials and Methods

Several national Finnish health and social welfare registers were utilized in this study, which enable comprehensive follow‐up of diagnosed diseases, treatments, and social benefits. These registers are maintained by the national authorities and can be linked to each other by personal identity codes. The Care Register for Health Care, maintained by the National Institute of Health and Welfare, includes information on inpatient care and specialized outpatient care visits. Registers from the Social Insurance Institution (SII) included sickness absences and disability pensions (identified from registers of SII and Finnish Centre for Pensions), which were utilized for the identification of persons with diagnosed depression, in addition to the Care Register for Health Care. Sickness absences included sick leaves of ≥14 days. Sick leaves and disability pensions also included diagnoses from primary care and the private sector. These registers, together with Earnings and Accrual registers provided information on the main occupational activity. Medication purchases were derived from two sources maintained by the SII: Prescription Register (since 1995, covering only reimbursed dispensings) and Kanta Services (covering all electronic prescriptions and dispensings, data was available since 2015). Special Reimbursement Register (maintained by the SII) contains information on granted special reimbursement for medications due to specific chronic conditions.

The base cohort of this study included all persons diagnosed with depression (International Classification of Diseases ICD‐10 F32‐F33) in inpatient care or specialized outpatient care, sickness absence or disability pension registers at the age of 16–65 years during the years 2015–2018 (*n* = 177 251).

### 2.1 Study Design

Inclusion criteria for this study were initiation of BZDRs between July 1, 2015, and June 30, 2018, with a diagnosis of depression recorded during the previous 90 days (*n* = 13,501). The 90‐day’ time frame for depression diagnosis was chosen to ensure that BZDR initiations most likely relate to depressive episode, instead of other or comorbid psychiatric conditions. Initiators (i.e., new users) were defined as those not having used BZDRs during the previous year before initiation. The index date was the first dispensing of a BZDR.

BZDRs were defined according to Anatomical Therapeutic Chemical (ATC) categories N05BA, N05CD (benzodiazepines), and N05CF (nonbenzodiazepines, i.e., Z‐drugs). The Prescription register and Kanta Services data on dispensed drugs included ATC‐code, dispensed amount, dispensing date, package size, and strength. Drug dispensing data were converted to drug use periods using the “from prescription drug purchases to drug use periods” (PRE2DUP) method (Tanskanen et al., 2015). Drug use periods are estimated time periods of when drug use started and ended based on dispensing dates, amounts dispensed, and drug‐specific parameters. The method is guided by expert‐defined parameters designed for each drug package, defining the minimum and maximum allowed doses and refill lengths. The method also takes into account stockpiling, days spent in hospital care, and the regularity of personal use. Drug use periods were constructed separately for each drug and the duration of “any BZDR” use was derived by combining overlapping drug use periods of all specific benzodiazepines and related drugs.

**FIGURE 1:**
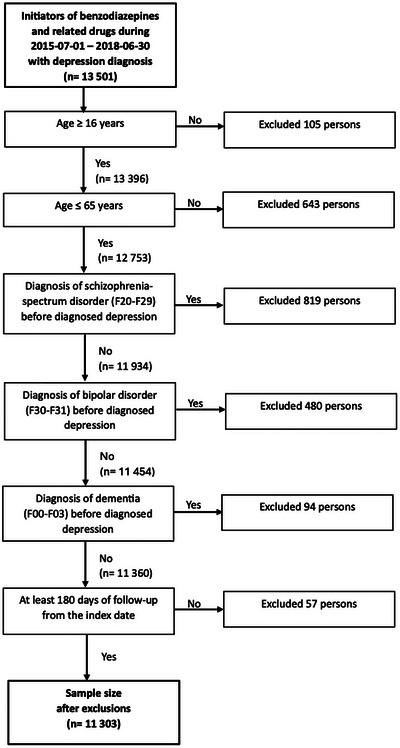
Flowchart of the exclusions.

Persons who were not in the age group 16–65 years at the index date, who had a diagnosis of bipolar disorder (F30–F31), schizophrenia‐spectrum disorder (F20–F29) or dementia (F00–F03) before initiation and those who did not have at least 180 days of follow‐up since the index date were excluded from the study (Figure 1). The final study sample after exclusions consisted of 11,303 persons (representing 0.33% of the Finnish population aged 16–65 years).

The primary outcome was long‐term use of benzodiazepines and related drugs. In this study, long‐term use of BZDRs was defined as 180 days or more of continuous use (no breaks, i.e., time periods of non‐use, in use after initiation allowed), as 180 days has been found to be the most common definition of long‐term BZDR use in previous studies (Kurko et al. 2015).

Sensitivity analyses were conducted by allowing a gap of 364 days in use and modeling time to long‐term use. The analyses were censored to death, diagnosis of schizophrenia‐spectrum disorder, bipolar disorder, or dementia, or at the end of data linkage, whichever occurred first.

### 2.2 Covariates

Covariates were measured before the index date, which was the first dispensing of BZDR (start of follow‐up). Detailed definitions of covariates are presented in Table S1. Age and gender were received from the SII. Socioeconomic activity was measured within 1 year before the start of follow‐up based on the Earnings and Accrual registers, supplemented with sickness absences and disability pensions. Severity of depression was defined as the most recent diagnosis before the index date and was based on diagnoses recorded in the Care Register for Health Care and by supplementing diagnosis data from the Special Reimbursement Register. The number of antidepressants (ADs) was categorized into four categories: 0, 1, 2, and ≥3 different drugs used in the previous year. Comorbidities included attention deficit hyperactivity disorder (ADHD), anxiety disorder, suicidality, smoking tobacco products, substance use disorder (including alcohol and other substances), active cancer, diabetes, inflammatory bowel diseases, and respiratory diseases, which were categorized dichotomously (yes/no). The use of antidepressants, that is, selective serotonin reuptake inhibitors (SSRIs), serotonin and norepinephrine reuptake inhibitors (SNRIs), tricyclic antidepressants (TCAs), mirtazapine, other ADs, ADs with hypnotic effects, was also defined at the start of follow‐up as a dichotomous variable (yes/no). The use of other drugs, that is, gabapentinoids, opioids, non‐opioid analgesics, quetiapine, melatonin, and hydroxyzine, was also defined dichotomously (yes/no).

### 2.3 Analyses

Descriptive statistics were used to analyze the demographic and socioeconomic variables (e.g., gender and employment status) of the study population, initiated BZDRs and the distribution of the variables was presented as frequencies (*n*) and percentages (%). The proportions of long‐term use of BZDRs were assessed as percentages and 95% confidence intervals (95% CI) based on initial BZDR type categorized as anxiolytic benzodiazepine (ATC N05BA) or hypnotic BZDR (N05CD, N05CF).

Odds ratios (OR) and 95% CIs were calculated with logistic regression to estimate potential predictors of long‐term use of BZDRs, using shorter‐term users as the reference group. Antidepressants were analyzed in a separate model as a higher number of antidepressants cannot be, by definition, reached without the use of several antidepressant classes. Also, as mirtazapine was conceptually a major part of the variable “antidepressants with hypnotic effects,” mirtazapine was not included separately in the adjusted model. In addition, pair‐wise Pearson correlation coefficients were calculated between all covariates to identify possible fair or moderate correlations, with a threshold of 0.30 (Table S2). This led to the exclusion of gabapentinoids due to a correlation with opioids (0.30%). The adjusted OR (aOR) with 95% CIs was visualized in the form of a forest plot.

A sensitivity model allowing gaps in use was analyzed with Cox regression as time to event. Unadjusted and adjusted hazard ratios (HRs) were calculated with 95% CIs (Table S3). Adjusted Cox regression models were performed using the same approach as logistic regression models described above: antidepressant variables were analyzed in a separate model, and mirtazapine was not included in either of the adjusted models.

Data management was performed using SAS version 9.4, and statistical analyses were performed using R version 4.3.3. and forest plot was created using R version 4.4.0.

## 3 Results

The mean age of initiators (*n* = 11,303) was 37.5 years (SD 13.0). Most of them were females (*n* = 7790, 68.9%) and nearly half were employed (*n* = 5446, 48.2%). The most frequent depression severity was moderate (*n* = 3966, 35.1%). Details of the baseline characteristics are shown in Table 1.

**TABLE 1:** Baseline characteristics of BZDR initiators with depression (*n* = 11,303).

	Sample size, *n* (%)
**Age, years**	
16–24	2367 (21.0)
25–34	2816 (24.9)
35–44	2421 (21.4)
45–54	2229 (19.7)
55‐	1470 (13.0)
**Gender**	
Male	3513 (31.1)
Female	7790 (68.9)
**Socioeconomic activity in the previous year**	
Employed	5446 (48.2)
Unemployed	1669 (14.8)
Sick leave	1419 (12.5)
Disability pension	1777 (15.7)
Parental leave	309 (2.7)
Other/unknown	686 (6.1)
**Severity of depression**	
Mild (F32.0, F33.0)	444 (3.9)
Moderate (F32.1, F33.1)	3966 (35.1)
Severe (F32.2, F32.3, F33.2, F33.3)	3015 (26.7)
Other (F32.8, F32.9, F33.8, F33.9)	3878 (34.3)

Abbreviations: BZDR, benzodiazepines and related drugs.

The most commonly initiated BZDR was oxazepam in both long‐term users (*n* = 374, 44.1%) and shorter‐term users (*n* = 4601, 44.0%), followed by zopiclone (*n* = 131, 15.4% for long‐term users and *n* = 1841, 17.6% for shorter‐term users) (Table 2). Two drugs were used at the same time by 366 (3.2%) persons, of whom 54 persons were long‐term users and 312 persons shorter‐term users. The most common combination was oxazepam and zopiclone (*n* = 15, 1.8% for long‐term users and *n* = 81, 0.8% for shorter‐term users) as well as oxazepam and temazepam (*n* = 17, 2.0% for long‐term users and *n* = 69, 0.7% for shorter‐term users). Three drugs were used at the same time by six (0.1%) persons and all of them were shorter‐term users. Long‐term users were more likely to use lorazepam (5.4% vs 2.8%), chlordiazepoxide (2.2% vs. 0.4%) and more than one BZDR at the same time (6.4% vs. 3.0%) than shorter‐term users. Shorter‐term users were more likely to use zopiclone (17.6% vs. 15.4%), temazepam (10.6% vs. 8.6%), and zolpidem (9.5% vs. 5.2%) than long‐term users.

**TABLE 2:** First BZDR initiated, compared to shorter‐term users with long‐term users.

	Long‐term users^a^ *n* = 849 *n* (%)	Shorter‐term users *n* = 10454 *n* (%)
Oxazepam (N05BA04)	374 (44.1)	4601 (44.0)
Zopiclone (N05CF01)	131 (15.4)	1841 (17.6)
Diazepam (N05BA01)	75 (8.8)	977 (9.3)
Temazepam (N05CD07)	73 (8.6)	1105 (10.6)
Lorazepam (N05BA06)	46 (5.4)	291 (2.8)
Zolpidem (N05CF02)	44 (5.2)	990 (9.5)
Alprazolam (N05BA12)	23 (2.7)	253 (2.4)
Chlordiazepoxide (N05BA02)	19 (2.2)	37 (0.4)
Other^b^	10 (1.2)	41 (0.4)
More than 1 drug	54 (6.4)	318 (3.0)

^a^ For at least 180 days, use of a BZDR.

^b^ Includes clobazam (N05BA09), nitrazepam (N05CD02), midazolam (N05CD08) and triazolam (N05CD05).

Abbreviation: BZDR, benzodiazepines and related drugs.

**FIGURE 2:**
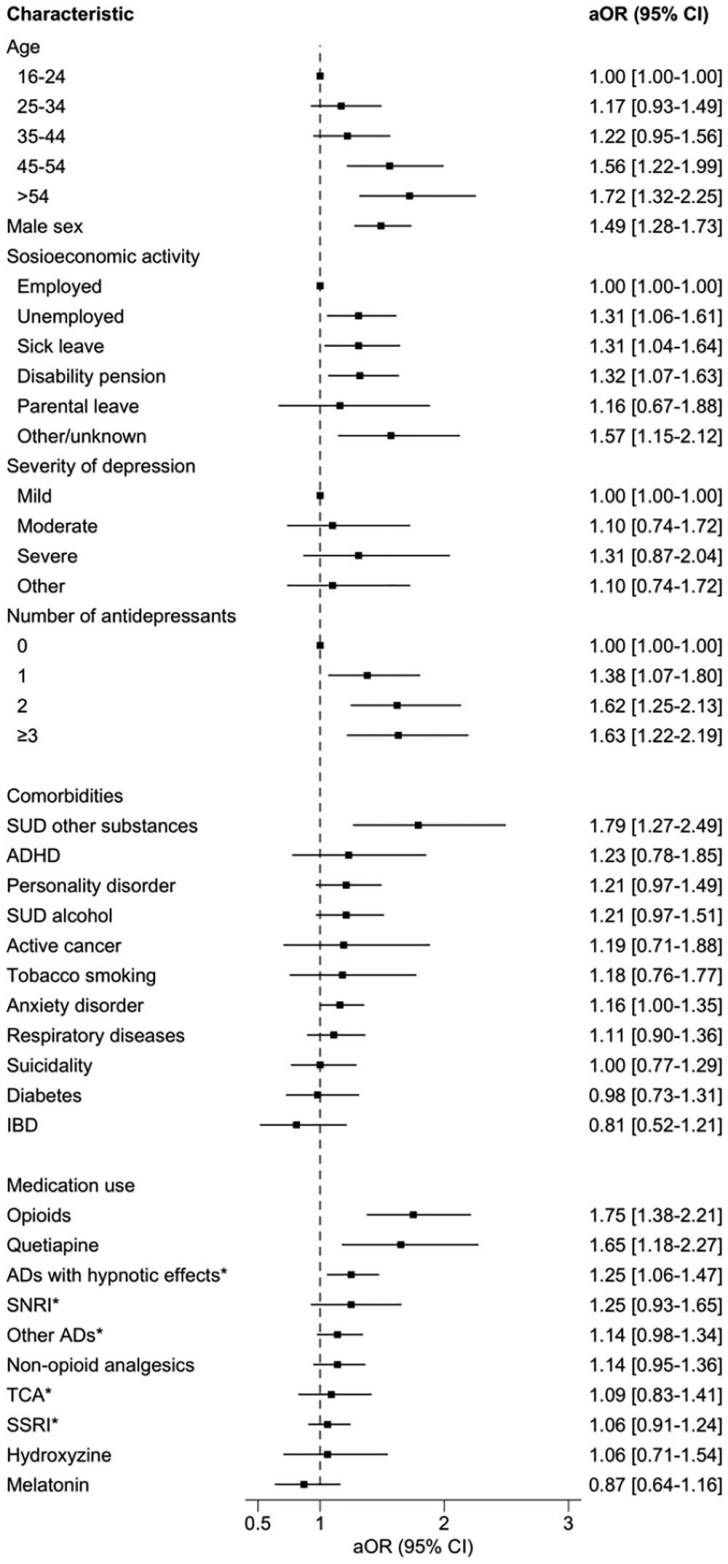
Factors associated with long‐term BZDR use among persons with depression, adjusted odds ratios from logistic regression model. The main adjusted model included all variables except the antidepressant subgroups (ADs with hypnotic effects, SNRI, other ADs, TCA, SSRI). *Included only in the second adjusted model, where the variable describing the number of antidepressants used in the previous year was removed because they were too highly correlated (≥0.30). Abbreviations: ADHD, attention deficit hyperactivity disorder; AD, antidepressant; aOR, adjusted odds ratio; CI, confidence interval; IBD, inflammatory bowel disease; SNRI, serotonin and norepinephrine reuptake inhibitor; SSRI, selective serotonin reuptake inhibitor; SUD, substance use disorder; TCA, tricyclic antidepressant.

Of the study population (*n* = 11 303), 849 (7.5%, CI 95% 7.0–8.0) persons met the criteria for long‐term use of BZDRs (Table 3). Of those initiating use with anxiolytic benzodiazepines, 8.5% (95% CI 7.8–9.1) became long‐term users, whereas 5.9% (5.2–6.7) of people initiating with hypnotic BZDRs became long‐term users. Mean age of long‐term users was 39.8 years (SD, 13.3). When compared to the youngest age group (16–24 years), the probability of long‐term use was higher among persons over 55 years (aOR, 95% CI 1.72, 1.32–1.73) and persons aged 45–54 years (1.56, 1.22–1.99) (Figure 2). Males had a higher risk of long‐term use (aOR 1.49, 95% CI 1.28–1.73) compared to females. Long‐term users were less often employed (*n* = 315, 37.1%) than shorter‐term users (*n* = 5131, 49.1%) (Table 3). Compared to employed persons, BZDR users who were unemployed (aOR 1.31, 95% CI 1.06–1.61), were on sick leave (1.31, 1.04–1.64) or on disability pension (1.32, 1.07–1.63) or had other/unknown socioeconomic status (1.57, 1.15–2.12) had higher risk of long‐term use (Figure 2).

**TABLE 3:** Characteristics of shorter‐term users compared with long‐term users of BZDRs.

	Long‐term users^a^ *n* = 849 *n* (%)	Shorter‐term users *n* = 10,454 *n* (%)	Unadjusted OR (95% CI)
**Age, years**			
16–24	144 (17.0)	2223 (21.3)	Ref.
25–34	190 (22.4)	2626 (25.1)	1.12 (0.89–1.40)
35–44	171 (20.1)	2250 (21.5)	1.17 (0.93–1.48)
45–54	202 (23.8)	2027 (19.4)	1.54 (1.23–1.92)
55	142 (16.7)	1328 (12.7)	1.65 (1.29–2.10)
**Gender**			
Female	500 (58.9)	7290 (69.7)	Ref.
Male	349 (41.1)	3164 (30.3)	1.61 (1.39–1.85)
**Socioeconomic activity in previous year**			
Employed	315 (37.1)	5131 (49.1)	Ref.
Unemployed	142 (16.7)	1527 (14.6)	1.51 (1.23–1.86)
Sick leave	127 (15.0)	1292 (12.3)	1.60 (1.29–1.98)
Disability pension	188 (22.1)	1589 (15.2)	1.93 (1.59–2.33)
Parental leave	17 (2.0)	289 (2.8)	0.96 (0.56–1.54)
Other/unknown	60 (7.1)	626 (6.0)	1.56 (1.16–2.07)
**Severity of depression**			
Mild	26 (3.1)	418 (4.0)	Ref.
Moderate	273 (32.2)	3693 (35.3)	1.19 (0.80–1.84)
Severe	283 (33.3)	2732 (26.1)	1.67 (1.21–2.58)
Other	267 (31.4)	3611 (34.6)	1.19 (0.80–1.84)
**Number of antidepressants used during the previous year**			
0	77 (9.1)	1483 (14.2)	Ref.
1	339 (39.9)	4529 (43.3)	1.44 (1.12–1.87)
2	277 (32.6)	2906 (27.8)	1.84 (1.42–2.40)
≥3	156 (18.4)	1536 (14.7)	1.96 (1.48–2.61)
**Psychiatric comorbidities**			
ADHD	25 (2.9)	238 (2.3)	1.30 (0.84–1.94)
Anxiety disorder	385 (45.3)	4187 (40.1)	1.24 (1.08–1.43)
Attempted suicide or other intentional self‐harm	83 (9.8)	771 (7.4)	1.36 (1.07–1.72)
Personality disorder	132 (15.5)	1150 (11.0)	1.50 (1.22–1.80)
Smoking (tobacco products)	26 (3.1)	221 (2.1)	1.46 (0.95–2.17)
Substance use disorder			
Alcohol	128 (15.1)	1005 (9.6)	1.67 (1.36–2.03)
Other substances	51 (6.0)	258 (2.5)	2.53 (1.84–3.41)
**Other comorbidities**			
Active cancer	20 (2.4)	169 (1.6)	1.47 (0.89–2.29)
Diabetes	58 (6.8)	525 (5.0)	1.39 (1.04–1.82)
Inflammatory bowel disease	25 (2.9)	326 (3.1)	0.94 (0.61–1.39)
Respiratory diseases (asthma, COPD)	124 (14.6)	1340 (12.8)	1.16 (0.95–1.41)
**Drug use at the start of follow‐up**			
SSRI	355 (41.8)	4664 (44.6)	0.89 (0.77–1.03)
SNRI	62 (7.3)	570 (5.5)	1.37 (1.03–1.78)
TCA	72 (8.5)	650 (6.2)	1.40 (1.08–1.79)
Mirtazapine	166 (19.6)	1644 (15.72)	1.30 (1.09–1.55)
Other antidepressants	314 (37.0)	3328 (31.8)	1.26 (1.09–1.45)
Antidepressants with hypnotic effects^b^	226 (26.6)	2158 (20.6)	1.39 (1.19–1.63)
Quetiapine	49 (5.8)	296 (2.83)	2.10 (1.52–2.84)
Gabapentinoids	71 (8.4)	408 (3.9)	2.25 (1.72–2.90)
Melatonin	52 (6.1)	739 (7.1)	0.86 (0.63–1.14)
Hydroxyzine	31 (3.7)	323 (3.1)	1.19 (0.80–1.70)
Opioids	110 (13.0)	652 (6.2)	2.38 (1.80–2.76)
Non‐opioid analgesics	197 (23.2)	1873 (17.9)	1.38 (1.17–1.63)

^a^ For at least 180 days use of a BZDR.

^b^ Mirtazapine, mianserin, trazodone, agomelatine, and doxepin.

Abbreviations: ADHD, attention deficit hyperactivity disorder; BZDR, benzodiazepines and related drugs; CI, confidence interval; COPD, chronic obstructive pulmonary disease; OR, odds ratio; SNRI, serotonin and norepinephrine reuptake inhibitor; SSRI, selective serotonin reuptake inhibitor; TCA, tricyclic antidepressant.

For long‐term users, most had severe depression (*n* = 283, 33.3%), followed by moderate depression (*n* = 273, 32.2%). In the unadjusted analyses, severe depression (1.67, 1.21–2.58) was associated with a higher risk of long‐term use than mild depression. However, in the adjusted model, severity of depression was not associated with long‐term use of BZDRs (1.31, 0.87–2.04). The number of antidepressants used during the previous year was associated with increased risk of long‐term use of BZDRs, compared with those who had not used any antidepressants in the previous year (1.63, 1.22–2.19 for ≥ 3 ADs, 1.62, 1.25–2.13 for two ADs, and 1.38, 1.07–1.80 for one AD).

Most common psychiatric comorbidities were anxiety disorder, which was present in 385 (45.3%) of long‐term users (*n* = 849), followed by personality disorder in 132 (15.5%) persons and alcohol use disorder in 128 (15.1%) persons. Persons with substance use disorder of substances other than alcohol (1.79, 1.27–2.49) and those with anxiety disorder (1.16, 1.00–1.35) had a higher risk of long‐term use.

Among the long‐term users of BZDRs, the most commonly used antidepressant was SSRI (*n* = 355, 41.8%). Antidepressants with hypnotic effects (including mirtazapine, mianserin, trazodone, agomelatine, and doxepin, *n* = 226) were used by 26.6% and mirtazapine specifically by (*n* = 166) 19.6%. Antidepressants with hypnotic effects were associated with increased risk of long‐term BZDR use (1.25, 1.06–1.47).

In the group of long‐term BZDR users, the most common use of other drugs was non‐opioid analgesics (*n* = 197, 23.2%) and opioids (*n* = 110, 13.0%). Opioids were associated with increased risk of long‐term use of BZDRs (1.75, 1.38–2.21). In addition, quetiapine use also increased the risk (1.65, 1.18–2.27) of prolonged use.

In a sensitivity analysis that allowed a gap of 364 days in use, 1357 (12%, CI 95% 11.4–12.6) persons met the criteria for long‐term use of BZDRs. The results of the Cox regression model (Table S3) were mostly consistent with the results of main analysis (Figure 2). The exceptions were that alcohol use disorder (1.26, 1.07–1.48), use of SNRIs (1.23, 1.00–1.52), and use of gabapentinoids (1.27, 1.03–1.58) were associated with an increased risk of long‐term use of BZDRs, although these were not statistically significant in the main analysis.

## 4 Discussion

In this nationwide population‐based study, 7.5% (CI 95% 7.0–8.0) of BZDR initiators with depression met the criteria for long‐term use. Incidence of long‐term use was relatively low compared to studies conducted in the general population. For example, a previous Finnish study reported 39% of BZDR initiators become long‐term users (Taipale et al. 2020). However, the study also included older people with a higher incidence (54.5%), whereas 33.8% in working working‐aged population met the criteria for long‐term use of BZDRs. Furthermore, the data used in the referenced study involve persons who initiated BZDR use in 2006, whereas our study utilized data from 2015 to 2018, and therefore studies represent a different time context. Over the past decade, prescribing practices for BZDRs have changed, the introduction of electronic prescriptions has improved monitoring of use, and alternative treatments for insomnia have become more widely available, such as low‐dose doxepin, low‐dose mirtazapine, and melatonin (Finnish Medical Society Duodecim and Finnish Sleep Research Society 2023). Our sensitivity model estimate (12%), allowing a maximum gap of 364 days in use, is methodologically closer to the previous study estimate (Taipale et al. 2020). However, the follow‐up time in the present study (a maximum of 3 years in relation to register linkage) was considerably shorter than in the previous study (9 years), which may also impact the difference in incidence estimates. To our knowledge, no previous study has assessed the risk of long‐term use in patients with depression.

Compared to other age groups, the risk of long‐term use was highest in those aged 55 years and older, but the risk was also increased in those aged 45–54 years. This is consistent with previously published studies showing that the prevalence of hypnotic drug use increases with age (Nygren et al. 2024) and that insomnia is more common in older adults compared to younger adults (Ohayon 2002).

A higher proportion of BZDR initiators were females. Previous studies have found that the use of hypnotic drugs in persons with depression was more common in females than in males (Nygren et al. 2024), and long‐term use of BZDRs was also more common in females in the general population (Kurko et al. 2018). Although females were more likely to initiate BZDR use, males had a higher risk of prolonged BZDR use than females. This is in line with previous findings where males were found to be more likely to become long‐term users of BZDRs (Taipale et al. 2020) and to use BZDRs at very high doses (≥3 defined daily doses/day) more frequently than females (Särkilä et al. 2024).

BZDR users who were unemployed, were on sick leave or on disability pension, or had other/unknown socioeconomic status had a higher risk of prolonged BZDR use compared to those who were employed. Similar findings have also been presented in a previous study, where receipt of social benefits was found to be associated with long‐term use of BZDRs in the general population (Taipale et al. 2020). Unemployment has been found to increase the risk of both depression (Zuelke et al. 2018) and insomnia (Blanchflower and Bryson 2021), which may partly explain the increased risk of long‐term BZDR use observed among persons with depression.

Nygren et al. (2024) reported in a recent study that persons with severe depression or a history of attempted suicide have a higher prevalence of hypnotic drug use. In the present study, the severity of depression was not associated with the long‐term use of BZDRs. However, a higher number of antidepressants used in the previous year was associated with increased risk of long‐term use compared with those who had not used any antidepressants in the past year. Use of three or more antidepressants may indicate treatment‐resistant depression, which is often defined as three consecutive treatment trials without adequate response (Hägg et al. 2020). Thus, it is possible that in this study, the severity of depression was more accurately described by the number of antidepressants used in the previous year than by the ICD‐10‐based classification of depression.

Among comorbidities, the highest risk of long‐term use was observed in persons with a diagnosis of substance use disorder other than alcohol. Similar results were found in a recent Danish study, where it was observed that among psychiatric comorbidities, substance use disorder in particular was associated with a high risk of long‐term BZDR use (Rosenqvist et al. 2024). A study by Nygren et al. (2024) also found similar results; among patients with a comorbid psychiatric diagnosis, those with substance use disorders (non‐alcohol/mixed) had the highest prevalence of hypnotic use.

Also, persons with anxiety disorder had an increased risk of long‐term use of BZDRs. Nordfjærn et al. (2014) found that anxiety was positively associated with chronic benzodiazepine use, supporting the observation that comorbid anxiety disorder may contribute to prolonged BZDR use also in depressed persons. Furthermore, a systematic literature review by Alvaro et al. (2013) found a bidirectional association between sleep disturbance, anxiety syndrome, and depression. This may indicate that these disorders may interact with each other and exacerbate symptoms.

Oxazepam was the most commonly used BZDR among both long‐term and short‐term users. The approved indication for oxazepam is the treatment of anxiety, but the Finnish Insomnia Current Care Guideline (2023) also recommends its short‐term use for insomnia. Initiation with lorazepam, chlordiazepoxide, or more than one BZDR was associated with increased risk for long‐term BDZR use, while initiation with zopiclone, temazepam, or zolpidem was associated with the lowest risk for prolonged use. Similarly, the proportion of long‐term users was lower when starting with hypnotic than anxiolytic BZDRs. Consistent with our findings, a previously published Finnish study found that starting treatment with lorazepam or polytherapy was associated with a higher risk of BZDR long‐term use, and initiation with Z‐drugs was associated with the lowest risk for long‐term use (Taipale et al. 2020).

The use of antidepressants with hypnotic effects and quetiapine, which may be used to treat insomnia, was associated with an increased risk of long‐term BZDR use. This may indicate that alternative drugs for the treatment of insomnia have been used before BZDR initiation. The use of BZDRs is not the first‐line preferred treatment for prolonged insomnia (Finnish Medical Society Duodecim and Finnish Sleep Research Society 2023; Riemann et al. 2023; Morin et al. 2024), so it is plausible that other sleep promoting drugs were trialed before BZDRs. Thus, quetiapine and antidepressants with hypnotic effects likely serve as proxies for more severe, chronic, or even treatment‐resistant insomnia.

Opioid use was also found to be associated with an increased risk of long‐term BZDR use. Chronic pain is common in patients with depression, and it can reduce sleep quality and exacerbate depressive symptoms (Karimi et al. 2023). One study found that more than 40% of those with depressive disorder also had a chronic painful physical condition (Ohayon and Schatzberg 2003). Opioids can be used to treat pain (Finnish Medical Society Duodecim et al. 2017). Pain symptoms and their treatment with opioids may partly be associated with prolonged BZDR use, especially in situations where pain impairs sleep and exacerbates depressive symptoms. However, it is somewhat concerning as concomitant use of BZDRs and opioids is associated with risks of sedation, respiratory depression and overdoses (Tori et al. 2020).

Main strengths of the study are nationwide data representing all BZDR initiators with depression. The national registers of health and social care covered diagnosed diseases, treatments, and social benefits. The study covered all persons aged 16–65 years who were diagnosed with depression in a specialized outpatient or inpatient care, or who were on sick leave or disability pension because of depression during the years 2015 and 2018. The register‐based analysis reduces the risk of selection bias and allows for a comprehensive review of the whole Finnish population. In addition, the study used the validated PRE2DUP method, which allowed the estimation of drug use periods based on dispensing dates, amounts dispensed, and drug‐specific parameters (Tanskanen et al. 2015; Forsman et al. 2018).

Limitations of the study are that the register data do not provide information on family and social characteristics, such as social support. We also lacked information on psychosocial treatments such as cognitive behavioral therapy for insomnia, CBT‐I, which is one of the main treatments for insomnia and has also been shown to reduce the use of sleep medication (Luik et al. 2020). We do not know to what extent the recommendations on non‐pharmacological treatments are followed in practice. Furthermore, in the depression severity classification, about one‐third of the cohort was classified as “other/unknown,” limiting the ability to estimate accurately the association between depression severity and long‐term use of BZDRs. In this study, the number of antidepressants used during the previous year likely described severity or treatment resistance of depression better than diagnosis‐based severity. Register‐based data may lack clinically important information, such as severity of sleep problems, and confounding by indication cannot be ruled out. Last, we lacked the actual clinical indications for the BZDR prescriptions.

## 5 Conclusion

In conclusion, the risk factors associated with long‐term use of BZDRs in this study were age over 45 years, male gender, substance use disorder other than alcohol, use of opioids or quetiapine, higher number of antidepressants used in the previous year, socioeconomic status other than employed, use of antidepressants with hypnotic effects, and anxiety disorder. These identified sociodemographic and clinical factors should be considered in the initiation and monitoring of BZDR treatment.

## Author Contributions

Concept: Heli Laitinen and Heidi Taipale. Analytic design: Heli Laitinen, Heidi Taipale, Miia Tiihonen, and Marjaana Koponen. Data analysis: Heli Laitinen, Heidi Taipale, and Antti Tanskanen. Data access and verification of data: Heli Laitinen and Heidi Taipale. First draft: Heli Laitinen. All authors contributed to interpreting the results and jointly decided to submit the manuscript for publication.

## Funding

The authors have nothing to report.

## Conflicts of Interest

Dr. Heidi Taipale, Dr. Jari Tiihonen, and Antti Tanskanen have participated in research projects funded by grants from Janssen to their employing institution. Dr Heidi Taipale reports personal fees from Gedeon Richter, Janssen, Lundbeck and Otsuka.  Dr. Jari Tiihonen has been a consultant and/or advisor to and/or has received honoraria from: Healthcare Global Village, HLS Therapeutics, Janssen‐Cilag, Lundbeck, Orion Pharma, Otsuka, Teva, and WebMD Global.

## Ethics Statement

The research project was approved by institutional authorities at the Social and Health Data Permit Authority FinData (THL/5279/14.06.00/2023), the Finnish National Institute for Health and Welfare (permission 635/5.05.00/2019), the Social Insurance Institution of Finland (31/522/2019, 160/522/2020), Finnish Centre for Pensions (19023) and Statistics Finland (TK‐53‐569‐19).

## Supporting information




**Supplementary Material**: brb371085‐sup‐0001‐SuppMat.docx

## Data Availability

Data collected for this study is proprietary of the Finnish government agencies Social Insurance Institution of Finland and the National Institute for Health and Welfare, which granted researchers permission and access to data. The data that support the findings of this study are available from these authorities, but restrictions apply to the availability of these data. The code used to analyze these data is available upon request by the corresponding author for purposes of reproducing the results.
