# Supplementary material for: Long‐Term Use of Benzodiazepines and Related Drugs in Persons With Major Depressive Disorder
Source: Brain Behav. 2025 Nov 17;15(11):e71085. doi: 10.1002/brb3.71085 (PMC12623834; doi:10.1002/brb3.71085)
Supplement: Supplementary file 1 — Supplementary Material: brb371085‐sup‐0001‐SuppMat.docx [file BRB3-15-e71085-s001.docx]

**Supplementary Table 1.** Definitions and classifications of baseline covariates.

| **Variable** | **Classification/definition**  **(ATC code, ICD-10, ICPC2 or NOMESCO codes, SR code)** | **Measurement point/ Time period** | **Data source** |
| --- | --- | --- | --- |
| Age, years | 16-24  25-34  35-44  45-54  55- | At the start of follow-up | SII |
| Gender | Male  Female | At the start of follow-up | SII |
| Sosioeconomic activity in the previous year | Employed  Unemployed  Sick leave  Disability pension  Parental leave  Student  Other/unknown | Within one year before start  of follow-up | EAR, SII |
| **Previous treatment of depression** |  |  |  |
| Severity of depression | Mild (ICD-10 F32.0, F33.0)  Moderate (ICD-10 F32.1, F33.1)  Severe (ICD-10 F32.2, F32.3, F33.2, F33.3), SR-code 112  Other (ICD-10 F32.8, F32.9, F33.8, F33.9) | Latest before the start of follow-up (ICD-10); or from 1972 to the start of follow-up (SR) | CRH, SRR |
| Number of antidepressants used during the previous year | 0  1  2  ≥ 3 | Within one year before start  of follow-up | PR, KS |
| **Psychiatric comorbidities** |  |  |  |
| ADHD | ICD-10 F90  SR-code 331 | From 1996 to the start of follow-up (ICD-10); or from 1972 to the start of follow-up (SR) | CRH, SRR |
| Anxiety disorder | ICD-10 F40-F43 | From 1996 to the start of follow-up | CRH |
| Attempted suicide or other intentional self-harm | ICD-10 X60-X84  ICD-10 Y10-Y34 (undetermined intent) | From 1996 to the start of follow-up | CRH |
| Personality disorders | ICD-10 F60-F61 | From 1996 to the start of follow-up | CRH |
| Smoking (tobacco products) | ICD-10:  F17 Mental and behavioural disorders due to use of tobacco  K03.61 Tobacco-induced deposits (accretions) on teeth  K05.31 Chronic periodontitis  K13.21 Tobacco-induced leukoplakia  K13.24 Smoker palate  Z71.6 Tobacco abuse counselling  Z72.0 Tobacco use (unless dependent)  ICPC2:  P17 Tobacco abuse  Drugs used in nicotine dependence: ATC N07BA (includes nicotine, varenicline), Zyban (bupropion) | From 1996 to the start of follow-up | CRH, PR |
| Substance use disorders | **Alcohol**  ICD-10:  F10 Mental and behavioural disorders due to use of alcohol  E51.2 Wernickes encephalopathy  E24.4 Alcohol-induced pseudo-Cushing syndrome  G31.2 Degeneration of nervous system due to alcohol  G40.51 Special epileptic syndromes  G62.1 Alcoholic polyneuropathy  G72.1 Alcoholic myopathy  I42.6 Alcoholic cardiomyopathy  K29.2 Alcoholic gastritis  K70 Alcoholic liver disease  K85.2 Alcohol-induced acute pancreatitis  K86.0 Alcohol-induced chronic pancreatitis  O35.4 Maternal care for (suspected) damage to fetus from alcohol  X45 Poisoning or other exposure to alcohol  Y91.0-91.3 Various degrees of alcohol poisoning  ICPC2:  P15 Chronic alcohol abuse  P16 Acute alcohol abuse  **Other substances**  ICD-10:  F11-F16, F18-F19  ICPC2:  P19 Drug abuse | From 1996 to the start of follow-up | CRH |
| **Other comorbidities** |  |  |  |
| Active cancer | ICD-10: C00-C97, or surgical  procedures related to cancer,  NOMESCO: AAG50, AX, HA0, PJO, QA0, QB0, QC0, QD0, QW0, QX0, WA, WB, WC, WD, WE, WF0, WFO, ZX0 | Within one year before start  of follow-up | CRH |
| Diabetes | ICD-10 E10-E14  SR-code 103, 215 | From 1996 to the start of follow-up (ICD-10); or from 1972 to the start of follow-up (SR) | CRH, SRR |
| Inflammatory bowel diseases | ICD-10 K50-52  SR-code 208 | From 1996 to the start of follow-up (ICD-10); or from 1972 to the start of follow-up (SR) | CRH, SRR |
| Respiratory diseases (Asthma, COPD) | ICD-10 J43-J45  SR-code 203 | From 1996 to the start of follow-up (ICD-10); or from 1972 to the start of follow-up (SR) | CRH, SRR |
| **Drug use at the start of follow-up** |  |  |  |
| **Antidepressants** |  |  |  |
| SSRI | ATC N06AB | Within 2 weeks before start of  follow-up | PR, KS |
| SNRI | ATC N06AX15 (venlafaxine)  ATC N06AX17 (milnacipran)  ATC N06AX21 (duloxetine) | Within 2 weeks before start of  follow-up | PR, KS |
| TCA | ATC N06AA | Within 2 weeks before start of  follow-up | PR, KS |
| Mirtazapine | ATC N06AX11 | Within 2 weeks before start of  follow-up | PR, KS |
| Other antidepressants | ATC N06AX04 (trazodone)  N06AG, N06AX excluding mirtazapine and SNRIs | Within 2 weeks before start of  follow-up | PR, KS |
| Antidepressants with hypnotic effects | ATC N06AX11 (mirtazapine)  ATC N06AX03 (mianserin)  ATC N06AX05 (trazodone)  ATC N06AX22 (agomelatine)  ATC N06AA12 (doxepin) | Within 2 weeks before start of  follow-up | PR, KS |
| **Sedative antipsychotics** |  |  |  |
| Quetiapine | ATC N05AH03 | Within 2 weeks before start of  follow-up | PR, KS |
| **Other** |  |  |  |
| Gabapentinoids | ATC N02BF  (Excluding indication epilepsy:  ICD-10 G40, SR: 111, 182 since 1996) | Within 2 weeks before start of  follow-up | PR, KS |
| Melatonin | ATC N05CH01 | Within 2 weeks before start of  follow-up | PR, KS |
| Hydroxyzine | ATC N05BB01 | Within 2 weeks before start of  follow-up | PR, KS |
| Opioids | ATC N02A | Within 2 weeks before start of  follow-up | PR, KS |
| Non-opioid analgesics | N02BE01 (paracetamol),  M01A (NSAIDs) | Within 2 weeks before start of  follow-up | PR, KS |

Abbreviations: ADHD, Attention Deficit Hyperactivity Disorder; ATC, Anatomical Therapeutic Chemical; COPD, Chronic Obstructive Pulmonary Disease; CRH, Care Register for Healthcare; EAR, The Earnings- and Accrual Register; ICD, International Classification of Diseases; ICPC, International Classification of Primary Care; KS, Kanta Services; NOMESCO, Nordic Medico-Statistical Committee; NSAID, non-steroidal anti-inflammatory drug; PR, Prescription Register; SII, Social Insurance Institution; SNRI, Serotonin and Norepinephrine Reuptake Inhibitor; SR, Special reimbursement; SRR, Special Reimbursement Register; SSRI, Selective Serotonin Reuptake Inhibitor; TCA, Tricyclic Antidepressant

**Supplementary Table 2.** Pearson’s correlation matrix.


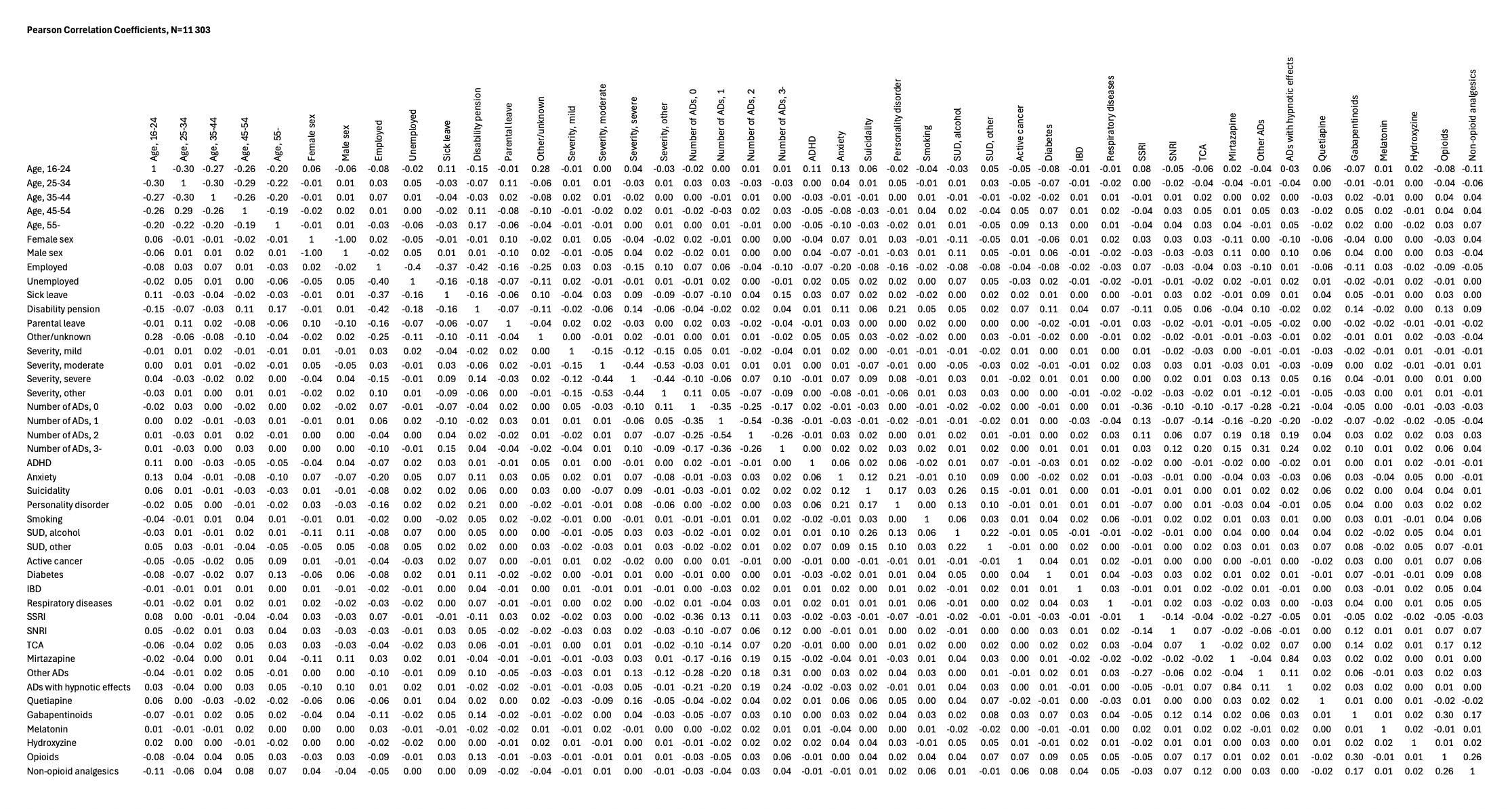


Abbreviations: AD, Antidepressant; ADHD, Attention Deficit Hyperactivity Disorder; IBD, Inflammatory bowel disease; SNRI, Serotonin and Norepinephrine Reuptake Inhibitor; SSRI, Selective Serotonin Reuptake Inhibitor; SUD, Substance use disorder; TCA, Tricyclic Antidepressant

**Supplementary Table 3. Characteristics of shorter-term users compared with long-term users of BZDRs, with maximum of gap of 364 days in use allowed and analyzed with Cox regression.**

|  | **Long-term users**^1^  **n=1357**  **n (%)** | **Shorter-term users**  **n=9946**  **n (%)** | **Unadjusted HR**  **(95 % CI)** | **aHR**  **(95 % CI)** |
| --- | --- | --- | --- | --- |
| **Age, years** |  |  |  |  |
| 16–24 | 242 (17.8) | 2125 (21.4) | Ref. | Ref. |
| 25–34 | 294 (21.7) | 2522 (25.4) | 1.00  (0.84–1.19) | 1.05 (0.88–1.26) |
| 35–44 | 274 (20.2) | 2147 (21.6) | 1.07  (0.90–1.28) | 1.10 (0.92–1.32) |
| 45–54 | 314 (23.1) | 1915 (19.2) | 1.34  (1.13–1.58) | 1.31  (1.09–1.57) |
| 55- | 233 (17.2) | 1237 (12.4) | 1.52  (1.27–1.82) | 1.56 (1.28–1.91) |
| **Gender** |  |  |  |  |
| Female | 820 (60.4) | 6970 (70.1) | Ref. | Ref. |
| Male | 537 (39.6) | (2976 (29.9) | 1.52  (1.36–1.69) | 1.39  (1.24–1.56) |
| **Socioeconomic activity in previous year** |  |  |  |  |
| Employed | 494 (36.4) | 4952 (49.8) | Ref. | Ref. |
| Unemployed | 235 (17.3) | 1434 (14.4) | 1.61  (1.38-1.88) | 1.40 (1.20-1.64) |
| Sick leave | 197 (14.5) | 1222 (12.3) | 1.61  (1.37-1.90) | 1.31 (1.10-1.55) |
| Disability pension | 310 (22.9) | 1467 (14.8) | 1.97  (1.71-2.28) | 1.35  (1.15-1.58) |
| Parental leave | 23 (1.7) | 283 (2.8) | 0.83  (0.54-1.25) | 1.00 (0.65-1.52) |
| Other/unknown | 98 (7.2) | 588 (5.9) | 1.63  (1.31-2.02) | 1.56  (1.24-1.96) |
| **Severity of depression** |  |  |  |  |
| Mild | 42 (3.1) | 402 (4.1) | Ref. | Ref. |
| Moderate | 432 (31.8) | 3534 (35.5) | 1.16  (0.85-1.59) | 1.10  (0.80-1.51) |
| Severe | 455 (33.5) | 2560 (25.7) | 1.68  (1.22-2.30) | 1.33  (0.96-1.83) |
| Other | 482 (31.6) | 3450 (34.7) | 1.18  (0.86-1.63) | 1.12  (0.81-1.53) |
| **Number of antidepressants used during the previous year** |  |  |  |  |
| 0 | 131 (9.7) | 1429 (14.4) | Ref. | Ref. |
| 1 | 518 (38.2) | 4350 (43.7) | 1.28  (1.06-1.55) | 1.23  (1.01-1.49) |
| 2 | 473 (34.9) | 2710 (27.2) | 1.85  (1.52-2.24) | 1.60  (1.32-1.95) |
| ≥ 3 | 235 (17.3) | 1457 (14.6) | 1.72  (1.39-2.13) | 1.40  (1.12-1.74) |
| **Psychiatric comorbidities** |  |  |  |  |
| ADHD | 38 (2.8) | 225 (2.3) | 1.26  (0.91-1.74) | 1.17  (0.85-1.63) |
| Anxiety disorder | 622 (45.8) | 3950 (39.7) | 1.26  (1.14-1.41) | 1.16  (1.04-1.31) |
| Attempted suicide or other intentional self-harm | 154 (11.35) | 700 (7.0) | 1.64  (1.39-1.94) | 1.18  (0.98-1.42) |
| Personality disorder | 212 (15.6) | 1070 (10.76) | 1.48  (1.28-1.71) | 1.15  (0.98-1.35) |
| Smoking (tobacco products) | 39 (2.9) | 208 (2.1) | 1.37  (0.99-1.88) | 1.05  (0.76-1.45) |
| Substance use disorder |  |  |  |  |
| Alcohol | 215 (15.8) | 918 (9.2) | 1.76  (1.52-2.04) | 1.26  (1.07-1.48) |
| Other substances | 78 (5.7) | 231 (2.3) | 2.36  (1.88-2.97) | 1.53  (1.20-1.96) |
| **Other comorbidities** |  |  |  |  |
| Active cancer | 29 (2.1) | 160 (1.6) | 1.30  (0.90-1.88) | 1.07  (0.74-1.56) |
| Diabetes | 96 (7.1) | 487 (4.9) | 1.44  (1.17-1.77) | 1.01  (0.82-1.25) |
| Inflammatory bowel disease | 50 (3.7) | 301 (3.0) | 1.18  (0.89-1.57) | 1.01  (0.76-1.34) |
| Respiratory diseases (asthma, COPD) | 202 (14.9) | 1262 (12.7) | 1.19  (1.03-1.38) | 1.10  (0.95-1.28) |
| **Drug use at the start of follow-up** |  |  |  |  |
| SSRI^2^ | 565 (41.6) | 4454 (44.8) | 0.89  (0.80-1.00) | 1.06  (0.94-1.19) |
| SNRI^2^ | 102 (7.5) | 530 (5.3) | 1.40  (1.14-1.71) | 1.23  (1.00-1.52) |
| TCA^2^ | 128 (9.4) | 594 (6.0) | 1.58  (1.32-1.90) | 1.20  (0.99-1.45) |
| Mirtazapine^4^ | 260 (19.2) | 1550 (15.6) | 1.28  (1.11-1.46) |  |
| Other antidepressants^2^ | 504 (37.1) | 3138 (31.6) | 1.26  (1.13-1.40) | 1.13  (1.00-1.28) |
| Antidepressants with hypnotic effects^2,3^ | 350 (25.8) | 2034 (20.45) | 1.34  (1.19-1.51) | 1.20  (1.06-1.36) |
| Quetiapine | 69 (2.8) | 276 (2.8) | 2.03  (1.59-2.58) | 1.51  (1.18-1.95) |
| Gabapentinoids | 112 (8.3) | 367 (3.7) | 2.22  (1.83-2.69) | 1.27  (1.03-1.58) |
| Melatonin | 101 (7.7) | 690 (6.9) | 1.16  (0.95-1.43) | 1.17  (0.96-1.44) |
| Hydroxyzine | 57 (4.2) | 297 (3.0) | 1.40  (1.08-1.83) | 1.23  (0.94-1.60) |
| Opioids | 184 (13.6) | 578 (5.8) | 2.33  (2.00-2.73) | 1.72  (1.44-2.05) |
| Non-opioid analgesics | 325 (23.9) | 1745 (17.5) | 1.41  (1.25-1.60) | 1.12  (0.98-1.29) |

^1^For at least 180 days use of a BZDR.

^2^Included only in the second adjusted Cox model, where the variable describing the number of antidepressants used in the previous year was removed due they were too highly correlated (≥ 0.30).

^3^Mirtazapine, mianserin, trazodone, agomelatine and doxepin.

^4^Excluded from the Cox model due it’s high correlation (≥ 0.30) with antidepressants with hypnotic effects variable.

Abbreviations: ADHD, Attention Deficit Hyperactivity Disorder; aHR, Adjusted Hazard Ratio; BZDR, Benzodiazepines and Related Drugs; CI, Confidence Interval; COPD, Chronic Obstructive Pulmonary Disease; HR, Hazard Ratio; SNRI, Serotonin and Norepinephrine Reuptake Inhibitor; SSRI, Selective Serotonin Reuptake Inhibitor; TCA, Tricyclic Antidepressant
